# Prevalence of Celiac Disease in Latin America: A Systematic Review and Meta-Regression

**DOI:** 10.1371/journal.pone.0124040

**Published:** 2015-05-05

**Authors:** Rafael Parra-Medina, Nicolás Molano-Gonzalez, Adriana Rojas-Villarraga, Nancy Agmon-Levin, Maria-Teresa Arango, Yehuda Shoenfeld, Juan-Manuel Anaya

**Affiliations:** 1 Center for Autoimmune Diseases Research (CREA), School of Medicine and Health Sciences, Universidad del Rosario, Carrera 24 #63-C-69, Bogotá, Colombia; 2 The Zabludowicz Center for Autoimmune Diseases, Sheba Medical Center, Tel-Hashomer, Israel; 3 Sackler Medical School, Tel Aviv University, Tel Aviv, Israel; 4 Incumbent of the Laura Schwarz-Kip Chair for Research of Autoimmune Diseases, Sackler Faculty of Medicine, Tel-Aviv University, Tel Aviv, Israel; 5 Doctoral Program in Biomedical Sciences Universidad del Rosario, Bogotá, Colombia; H. Lee Moffitt Cancer Center & Research Institute, UNITED STATES

## Abstract

**Background:**

Celiac disease (CD) is an immune-mediated enteropathy triggered by the ingestion of gluten in susceptible individuals, and its prevalence varies depending on the studied population. Given that information on CD in Latin America is scarce, we aimed to investigate the prevalence of CD in this region of the world through a systematic review and meta-analysis.

**Methods and Findings:**

This was a two-phase study. First, a cross-sectional analysis from 981 individuals of the Colombian population was made. Second, a systematic review and meta-regression analysis were performed following the Preferred Reporting Items for Systematic Meta- Analyses (PRISMA) guidelines. Our results disclosed a lack of celiac autoimmunity in the studied Colombian population (i.e., anti-tissue transglutaminase (tTG) and IgA anti-endomysium (EMA)). In the systematic review, 72 studies were considered. The estimated prevalence of CD in Latin Americans ranged between 0.46% and 0.64%. The prevalence of CD in first-degree relatives of CD probands was 5.5%. The coexistence of CD and type 1 diabetes mellitus varied from 4.6% to 8.7%, depending on the diagnosis methods (i.e., autoantibodies and/or biopsies).

**Conclusions:**

Although CD seems to be a rare condition in Colombians; the general prevalence of the disease in Latin Americans seemingly corresponds to a similar scenario observed in Europeans.

## Introduction

Celiac disease (CD) is an autoimmune intestinal disorder. This disease occurs due to an immune-mediated enteropathy triggered by ingested prolamins present in wheat, barley, and rye (generically called gluten). It occurs in susceptible individuals carrying the HLA-DQ2 and HLA-DQ8 haplotype [1].

Recently the American College of Gastroenterology and the British Society of Gastroenterology provided recommendations to perform an initial screen with IgA anti-tissue transglutaminase (tTG) [2,3]. Meanwhile, the ESPGHAN (European Society of Pediatric Gastroenterology and Nutrition) proposed various criteria for diagnosis over time. Initially, the diagnosis required a sequence of three small intestinal biopsies, but recently the guidelines indicated that symptomatic children with high levels of tTG and positive anti-endomysium (EMA) as well as HLA DQ2/8 do not need the biopsy for disease diagnosis [4–6]. IgA tTG and IgA EMA autoantibodies have a sensitivity and specificity of 98–100% [7]. They have been used for the CD diagnosis since the 1990s [8]. Antigliadin (AGA) and anti-reticulin antibodies have also been previously used, but they are currently considered obsolete for diagnosis because of their low sensitivity and specificity [9]. In brief, individuals that have tested positive for celiac autoantibodies are considered as potentially diagnosed patients, regardless of the biopsy results [10].

The distribution of CD has been associated with migratory patterns and changes in feeding habits over time. In the early years, humans were not exposed to gluten contained within cereals. Approximately 10,000 years ago in a small region of the Middle East, farmers successfully cultivated wild wheat and barley grains due to favorable environmental conditions. These people then migrated to the Mediterranean area (Northern Africa and Southern Europe) and Central Europe, searching for new lands to cultivate [11].

CD is a very frequent disorder in highly populated countries where inhabitants have a white ancestry, primarily in Europe and North America. However, CD has also been reported among people with Amerindian and African origins [12,13]. CD affects 0.6 to 1.0% of the population worldwide and exhibits a female-to-male ratio of 2.8:1 [14]. Also the age of onset distribution shows a first peak between nine months to two years and a second peak during the fourth decade [14]. The frequency of CD is likely to increase in many developing countries due to an increased prevalence of a “westernized” diet, involving greater wheat production. For instance, over the past 30 years, the prevalence of CD in the United States has increased five-fold, doubling approximately every 15 years [15].

The presence of CD in the Latin American (LA) population is uncertain. Latin America is the geographical area defined by Mexico, Central America, islands of the Caribbean, and South America. It is a rapidly growing region with almost 600 million inhabitants [16]. The LA population is mixed with ancestries including Africans, Caucasians, and Amerindians [17]. In the present study, we aimed to analyze the prevalence celiac autoimmunity (i.e., tTG and EMA) in a Colombian population as a surrogate of CD [18]. We evaluated the presence of these autoantibodies in healthy individuals and in patients with other autoimmune conditions, given that CD may coexist with and share similar immunopathological mechanisms with other autoimmune diseases (ADs) [19]. In addition, we performed a systematic literature review and meta-regression analysis to determine the estimated prevalence of CD in LA.

## Methods

### Study population

A total of 981 individuals from two regions in Colombia (Northwest and Central) were assessed for the presence of CD autoantibodies (Table 1). There were 541 affected and 140 unaffected individuals from Northwest Colombia (Group 1), whilst Group 2 (Central Colombia) was comprised of 180 AD patients and 120 non-AD controls, taken from an original cohort of 1667, and sampled according to a stratified sampling design where the strata were different cohorts of patients with ADs.

**Table 1:** Characteristics and results of the antibodies in two populations.

				Group 1 Northwest (Antioquia)				Group 2 Central (Cundinamarca and Boyacá)		
Age (mean±SD)				45,5 ± 12,75				41,15 ± 13,65		
Female				91,20%				90,33%		
Autoantibodies	CTR	SLE	RA	SS	MS	T1DM and relatives	CTR	SLE	RA	SS
	n = 140	n = 119	n = 151	n = 82	n = 98	n = 91*	n = 120	n = 60	n = 60	n = 60
IgA AGA	5	6	4	5	7	2	ND	ND	ND	ND
IgG AGA	2	12	10	6	10	17	ND	ND	ND	ND
IgA tTG	0	0	0	0	0	1**	0	1	4	2
IgG tTG	0	9	4	1	2	2	ND	ND	ND	ND
IgA EMA	ND	ND	ND	ND	ND	ND	0	0	0	0

* 67 children with T1DM and 24 first-degree relatives.

** T1DM Relative.

Abbreviations: AGA: Antigliadin antibody, CTR: Healthy controls, EMA: anti-endomysium antibody, MS: multiple sclerosis, ND: Not done, RA: rheumatoid arthritis, SS: Sjögren’s syndrome, SLE: systemic lupus erythematosus, T1DM: Type 1 diabetes mellitus, tTG: Transglutaminase tisular antibody.

All of the patients with ADs fulfilled the international classification criteria. These include: the American College of Rheumatology (ACR) criteria for RA and SLE, the McDonald criteria for MS and the American Diabetes Association (ADA) criteria for T1DM [20–23]. Patients with SS met the American-European Consensus Group criteria; including a positive minor salivary gland biopsy (MSGB) [24]. General and clinical characteristics of these patients have been previously described [25–46].

The information regarding patient demographics as well as cumulative clinical and laboratory data was obtained by physical examination, interviewing or chart reviews as described elsewhere [25–46]. All data were collected in an electronic and secure database. The review board and the ethics committee of Universidad del Rosario approved the study according to the ethical guidelines of the Helsinki Declaration and Resolution 008430 of 1993 of the Ministry of Health in Colombia. The study was classified as minimal risk research. All patients completed the written informed consent.

### Detection of Autoantibodies

In Group 1, IgA and IgG antibodies against AGA and tTG were assessed by the Bio-Rad BioPlex 2200 system (Bio-Rad Laboratories, Hercules, California, USA) as previously described [25,27,28,36–46].

In Group 2, IgA h-tTG (native human tissue transglutaminase) antibodies were assessed using the ELISA method (INOVA Diagnostic, USA. Cat. 708730) in the DYNEX DS2 ELISA analyzer. According to the manufactured instructions, samples were classified as negative (<20 units), weakly positive (20–30 units) or positive (>30 units). Weakly positive samples were analyzed by indirect immunofluorescence (IFI) assay to evaluate the presence of IgA EMA, using two additional commercial kits (INOVA Diagnostics, USA. Cat. 508154, and AESKU.Diagnostics, Germany. Cat. 512.050).

### Search strategy

The search was done using the following databases: PubMed, Cochrane, Scopus, SciELO, and Virtual Health Library, which includes BIREME, LILACS and many other LA sources. The search was related to CD in LA and included articles published up to July 2013. The PRISMA guidelines were followed during data extraction, analysis, and reporting [47].

No limits regarding language, publication type, or publication period were taken into account. The search was done with the following MeSH terms (Medical Subject Headings) and key words in PubMed, Scopus and Chocrane: *celiac disease*, *transglutaminase antibody*, *antigliadin antibody*, *gliadin antibody*, *deamidated gliadin antibody*, *endomysial antibody*, *anti endomysium antibody*, *HLA-DQ2* and *HLA-DQ8*. Each one was cross-referenced with the following MeSH terms: *Latin America*, *Hispanic Americans*, *Hispanics*, *South America*, *Argentina*, *Belize*, *Bolivia*, *Brazil*, *Chile*, *Colombia*, *Costa Rica*, *Cuba*, *Dominican Republic*, *Ecuador*, *El Salvador*, *Guatemala*, *Haiti*, *Honduras*, *French Guiana*, *Mexico*, *Nicaragua*, *Panama*, *Paraguay*, *Peru*, *Puerto Rico*, *Surinam*, *Uruguay*, and *Venezuela*.

The same methodology and the term *celiac disease* (without other cross terms) were used to explore sources of information in Spanish, Portuguese, and English through the SciELO and Virtual Health Library databases. Each MeSH term and keyword was translated into DeCS (Health Sciences Descriptors).

### Study selection, data extraction, and quality assessment

Inclusion criteria for the systematic review were the following: (a) studies with screening tests for autoantibodies of CD such as AGA, EMA, tTG, or deamidated gliadin peptide (DGP) in healthy individuals or in patients without CD diagnosis; (b) studies with screening tests for autoantibodies of CD and a positive biopsy in healthy individuals or in patients without CD diagnosis; (c) studies that include the LA population. Studies were excluded if they were reviews or case reports or if they discussed topics not related to CD. Unpublished data were also excluded. A primary reviewer who screened all of the titles and abstracts from the publications performed an eligibility assessment. Retrieved articles were rejected if the eligibility criteria were not met. Also, a secondary reviewer was consulted when eligibility criteria were unclear. References from the articles that seemed to be relevant for our review were hand-searched.

The extracted data from each article were: author name, country where the study took place, year of publication, study design, number of patients, screening protocol and evaluated outcomes. Several studies used different screening protocols to evaluate the presence of autoantibodies. Therefore, we considered the results to be positives based on the cutoff of each protocol. All articles were assessed according to the Oxford Center for Evidence based Medicine: 2011 Levels of Evidence [48].

### Meta-regression

A population variable was created which addressed the nature of the cases in each article: Population A: Healthy individuals; Population B: First-degree relatives of CD patients; Population C: T1DM (type 1 diabetes mellitus) patients; Population D: Patients with other ADs; Population E: Patients with other conditions. Studies published previously with the same population were excluded. The data obtained in the present study were involved in the meta-analyses.

During the search, several studies used different screening protocols to estimate the presence of CD. Taking this into account, we performed a meta-regression of the two forms of diagnostic protocols separately; the first set were studies that evaluated the presence of IgA tTG and EMA (sensitivity and specificity of 98–100%), and the second set were the studies that evaluated the positive autoantibodies and positive biopsy, following the statistical model-building approach described below.

The meta-analysis was performed for each diagnostic protocol separately, by fitting a meta-regression model with random effects and testing different combinations of predictor variables available in all articles: a) year of study, b) country and c) population (nature of cases in each study, as described previously) to explain the log-prevalence of the disease. The final predictors included in the two meta-regression models (one for each diagnostic protocol) were selected according to the likelihood ratio test and AIC criteria as described in [49]. Over the selected model, routine diagnostic tests of meta-analysis were performed (test for funnel plot asymmetry, *I* ^*2*^, H^2^, among others). The analysis was performed with the R2.15.2 package METAFOR [49].

## Results

### Colombian population

In Group 1, IgA tTG was positive only in one relative of a T1DM patient. In Group 2, seven individuals were positive or weakly positive for h-tTG using the reference values from the commercial Kit. In all of these cases, the evaluation of IgA EMA was negative (confirmed by two different kits). These results are shown in Table 1.

### Systematic Literature Review

**Fig 1:**
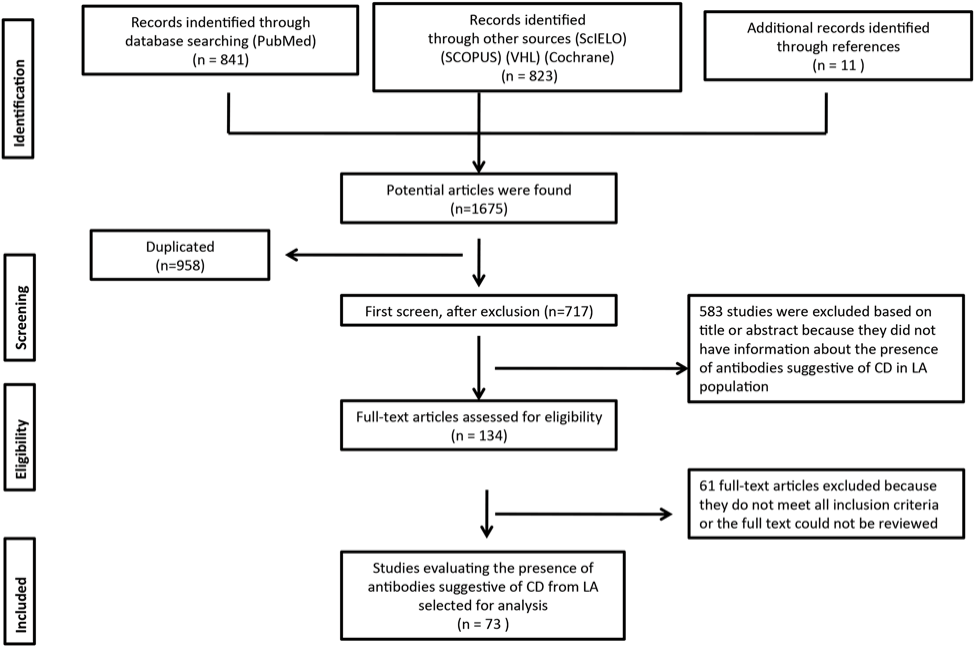
Flow chart of the systemic literature review. VHL: virtual health library. CD: Celiac disease. LA: Latin America.

We identified 841 articles in the PubMed database search. Additional records identified through other sources included 823 articles (Scopus, SciELO, Virtual Health Library and Cochrane). Eleven additional records were identified through hand searching. The database searches provided a total of 1,675 publications. Of these, 958 were identified as duplicates. A total of 717 full text articles were assessed for eligibility. Finally, 73 articles that contained interpretable data and fulfilled the eligibility criteria were included [50–122]. In one paper, the data extraction was made from its abstract [50]. Eight articles were from Argentina [51–58], 41 from Brazil [50,59–98], 3 from Chile [99–101], 11 from Cuba [102–112], 5 from Mexico [113–117], 2 from Peru [118,119], 2 from Venezuela [120,121] and one paper was from the Hispanic residents in the United States [122]. The flowchart for systematic literature review and articles included in the analysis is shown in Fig 1. Detailed information is shown in S1 Table.

### Meta-analysis

#### Meta-regression for tTG and EMA protocol

A total of 27 studies comprising 28 articles were included in the model [50,52,54,55,66,69,71–74,78,80,82,83,85–87,89,90,94,97,100,114,116–118,121,122]. The work of Remes-troche, *et al*. was analyzed as one study because they evaluate the presence of CD in the same population, but in two different time periods [114,117]. In five studies obtained from the systematic review, the population was divided into subpopulations [66,69,78,89,90]. In addition, our two subpopulations from the central area of Colombia were included in the analysis (i.e., healthy individuals and patients with ADs). In summary, 34 populations were analyzed (S1 Fig).

The most parsimonious model (according to the AIC criteria and likelihood ratio tests) was a meta-regression with random effects, which involves the population and country variables as the only moderators. The study from Venezuela was excluded due to low sample size and the high prevalence, and was therefore considered as an outlier [121]. Table 2 shows the estimated parameters of this model.

**Table 2:** Meta-regression model for tTG and EMA protocol.

	estimate	s.d.	pval	95% C.I.	
intrcpt (Brazil & Pop. A)	-5.0462	0.2455	<.0001	-5.5275	-4.565
Argentina	0.3878	0.4508	0.3897	-0.4958	1.2714
Chile	0.5883	1.4914	0.6932	-2.3348	3.5114
Colombia	-0.6819	1.1855	0.5652	-3.0053	1.6416
Mexico	0.1383	0.5408	0.7981	-0.9216	1.1983
Peru	0.0824	1.1771	0.9442	-2.2246	2.3895
USA	-2.7854	1.1725	0.0175	-5.0835	-0.4872
Population B	2.1509	0.3873	<.0001	1.3919	2.91
Population C	2.6059	0.4567	<.0001	1.7109	3.501
Population D	0.0759	0.9788	0.9382	-1.8425	1.9943
Population E	1.5298	0.3882	<.0001	0.7689	2.2907
estimated tau: 0.5612		I^2: 66.14%		H^2: 2.95	
Test for Residual Heterogeneity: p-val <.0001					
Test of Moderators: p-val <.0001					

(Prevalence in log-scale). Population: A: Healthy individuals; B: First-degree relatives of CD patients; C: T1DM patients; D: Patients with other ADs; E: Patients with other conditions.

**Fig 2:**
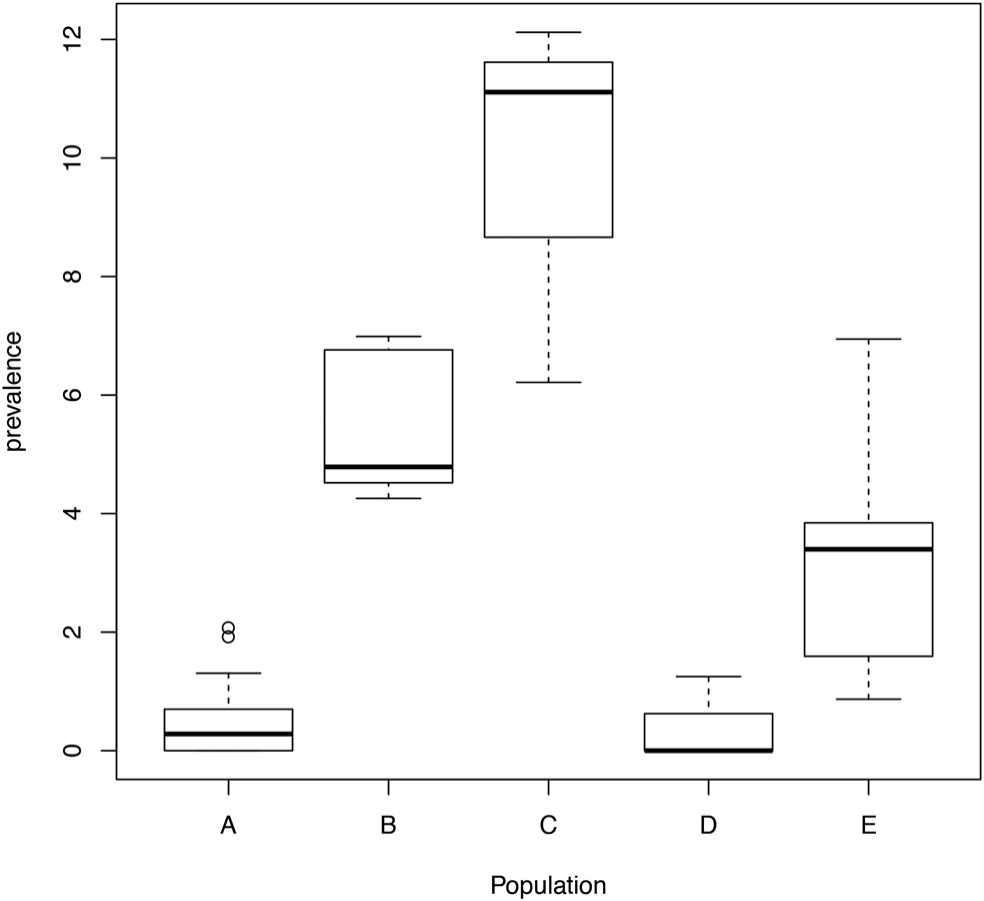
Boxplot of the prevalence of the disease for each population using the model for tTG and EMA protocol. Population: A: Healthy individuals; B: First-degree relatives of CD patients; C: T1DM patients; D: Patients with other ADs; E: Patients with other conditions.

The parameters associated with countries and populations refer to the log-prevalence between various country and population combinations when compared against Brazil, regarding population A (healthy individuals). The intercept parameter was the log-prevalence for Brazilian individuals regarding controls. Note that this Brazilian population was chosen as a reference level due to its large number of studies. Taking these parameters into account, only Hispanics from the USA had a log-prevalence less than that of the Brazilians, despite the different population groups that were analyzed. However, populations B, C and E had a higher prevalence of the disease than population A. Furthermore, the observed parameters of population D were not significant. This suggests that the prevalence of the disease is higher in individuals with first-degree relatives diagnosed with CD and T1DM (Fig 2 and S1 Fig).

We did not find any evidence of publication bias (test for funnel plot asymmetry: z = -1.8174, p = 0.0692). Additionally, two subpopulations in two different studies presented lack of fit to this model [64, 66] (S2 Table and S3 Fig). This discrepancy could be explained by the characteristic of the studied population (i.e., pediatric inpatients and outpatients).

#### Meta-regression for autoantibodies positive and biopsy positive protocol

A total of 49 studies were included in this model [50,52–55,58–73,75,77,79–81,83,85,88,90,94,96,99–102,104,107,109–113,116–121]. Within three studies obtained from the systematic review, the population was divided into subpopulations. As a result, 51 populations were analyzed.

Again, the most parsimonious model (according to the AIC criteria and likelihood ratio tests) was a meta-regression with random effects. As was the case with the previous diagnostic protocol, the only moderators in this model were the population and country variables. Therefore, interpretation of meta-regression parameters was the same as before. Table 3 shows the estimated parameters of the selected model.

**Table 3:** Meta-regression model for autoantibodies positive and biopsy positive.

	estimate	se	pval	95% C.I.	
intrcpt (Brazil & Pop. A)	-5.3769	0.2522	<.0001	-5.8712	-4.8827
Argentina	0.8688	0.3932	0.0271	0.0981	1.6394
Chile	0.0924	0.6638	0.8892	-1.2085	1.3934
Cuba	0.6998	0.3941	0.0757	-0.0725	1.4722
Mexico	0.4732	0.663	0.4753	-0.8261	1.7726
Peru	3.4203	0.5631	<.0001	2.3167	4.5239
Venezuela	-0.8973	0.8812	0.3086	-2.6244	0.8298
Population B	2.4953	0.4453	<.0001	1.6226	3.3681
Population C	2.2998	0.3999	<.0001	1.516	3.0835
Population D	0.9452	0.8359	0.2581	-0.693	2.5835
Population E	1.6394	0.3182	<.0001	1.0158	2.263
estimated tau: 0.7134		I^2: 78.90%		H^2: 4.74	
Test for Residual Heterogeneity: p-val <.0001					
Test of Moderators: p-val <.0001					

(Prevalence in log-scale). Population: A: Healthy individuals; B: First-degree relatives of CD patients; C: T1DM patients; D: Patients with other ADs; E: Patients with other conditions.

**Fig 3:**
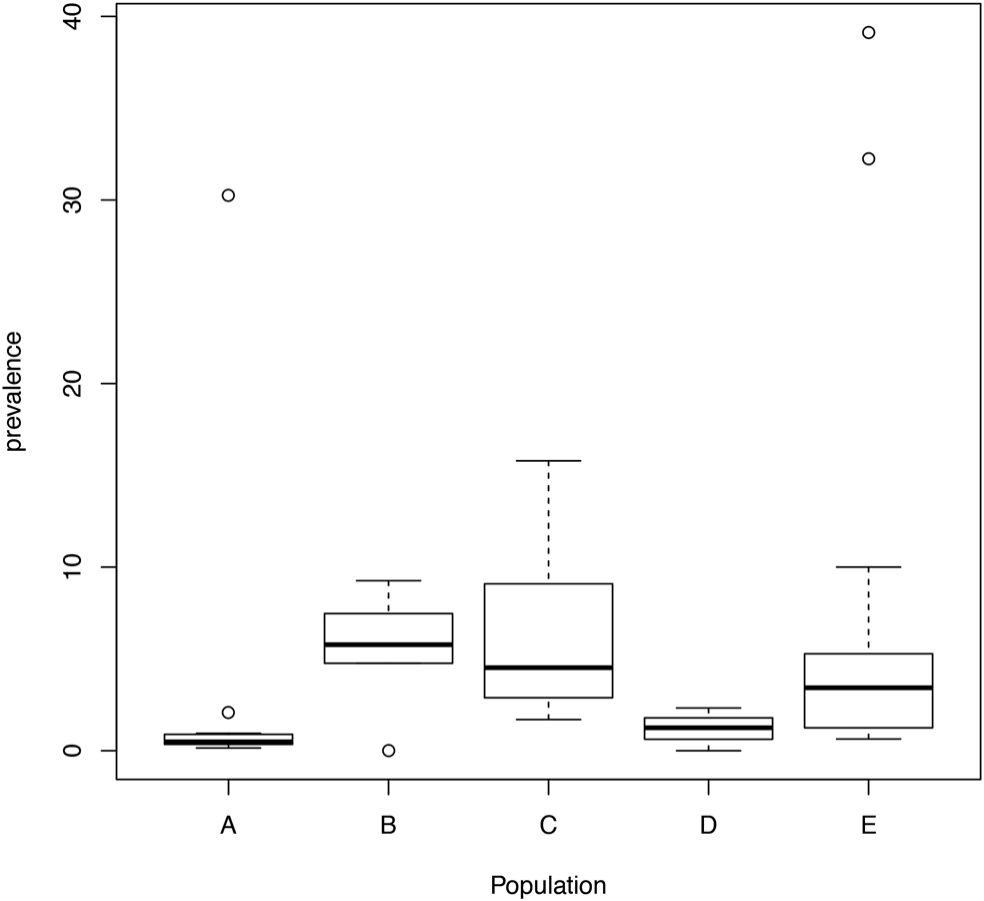
Boxplot of the prevalence of the disease for each population using the model for autoantibodies positive and biopsy positive. Population: A: Healthy individuals; B: First-degree relatives of CD patients; C: T1DM patients; D: Patients with other ADs; E: Patients with other conditions.

Populations from Peru and Argentina have a higher log-prevalence than Brazilian individuals, whilst populations B, C and E had a higher prevalence of the disease than population A. Furthermore, the observed parameters of population D were not significant. As previously found in the tTG and EMA protocol, the prevalence of the disease was higher in individuals with first-degree relatives diagnosed with CD and T1DM (Fig 3 and S2 Fig).

Note that we found evidence of publication bias (test for funnel plot asymmetry: z = -3.4489, p = 0.0006). Additionally, two subpopulations in two different studies exhibited a lack of fit to the model [58,66] (S3 Table and S4 Fig). This discrepancy could once again be explained by the characteristic of the studied population (pediatric inpatients and patients with clinical suspicion), as suggested in the meta-regression analysis for the tTG and EMA protocol.

## Discussion

Although CD seems to be a rare condition in Colombians; the general prevalence of the disease in Latin Americans seemingly corresponds to a similar scenario observed in Europeans (0.46% to 0.64%) [123,124]. Our study is the first to summarize and analyze all published studies about CD in the LA population. The presence of the disease was evaluated in a population with different characteristics, and we found 801 patients positive for IgA tTG and IgA EMA and 454 patients positive for autoantibodies and biopsy (S1 Table).

The LA population presents with a notable racial, genetic, and cultural diversity [17]. Therefore in LA, some countries such as Argentina, Brazil, Chile, Cuba, Uruguay and Venezuela, have > 50% of Caucasian component, and certain regions within these nations are more Caucasian than others [111,125]. The highest prevalence of CD has been reported in those countries with a high Caucasian admixture (S1 Table). However, in nations such as Chile, Uruguay and Venezuela, the prevalence of CD is uncertain. Brazil is the only country where the presence of CD has been widely studied. This country is an important migratory destination for European Caucasians. These immigrants are located primarily in the Southern region of Brazil, and most cases of CD have been reported in this region. In contrast, the North and the Northeast region of Brazil have large Amerindian and African ancestral influences respectively. In those regions the presence of the disease is low [126]. In addition, the presence of the disease in native Indian and African-derived communities from the Northeast Brazilian region is null [84,95] (S1 Table).

Our meta-regressions also show that Hispanics in the United States (i.e., a person of Mexican, Puerto Rican, Cuban, South or Central American (except for Brazil), or other Spanish culture or origin, regardless of race) have a different behavior than individuals of other countries. The prevalence of CD in Hispanic was estimated in 2.519 individuals (1.686 Mexican Americans and 833 as other Hispanic groups). Only one patient was positively detected by autoantibodies (IgA tTG and IgA EMA), whilst the other two patients were detected by interview data. However, none of these patients were Mexican-American [122]. Nevertheless, these results are very different from those observed in the Mexican population (Tables 1 and 2, S1 Table, Figs 2 and 3, and S1 and S2 Figs). Thus, this discrepancy in the prevalence on native Mexicans vs. Mexican Americans highlights the major effect of environmental factors over genetic factors in the risk of developing CD.

Polyautoimmunity (i.e., the presence of two or more ADs in a single patient) [19] was observed in our study. The estimated prevalence of CD in the T1DM patients was 4.6 to 8.7%. This number is similar to the prevalence reported in other populations [127]. The association among CD and T1DM can be explained by the presence of common risk alleles including HLA-DQA1*0501 and DQB1*0201 [128]. However, the estimated prevalence of CD in the group of patients with ADs was 0.7% to 1%. This surprisingly low prevalence may be explained by the small number of studies available and the lack of awareness about polyautoimmunity [98]. In fact, most of the studies evaluating this association came from case reports or from studies with a small sample size (S1 Table).

Both familial CD [129] and familial autoimmunity (i.e., the presence of diverse ADs in relatives of CD probands) are frequent conditions [130,131], indicating aggregation of the autoimmune trait. The prevalence of CD in first-degree and second-degree relatives is 10% and 5% respectively [132]. In our results, the estimated prevalence of CD in first-degree relatives was about 5.6%, whereas the estimated prevalence in populations with other conditions, such as at-risk populations, was 0.24% to 0.3% (S1 Table). This result could be explained by the heterogeneity of the study population and the small sample size.

Although the two Colombian population analyzed in this study may disclose a different genetic background [133], no celiac autoimmunity was observed in neither. Colombians in general still eat cultivated food, such as potatoes, tapioca and corn [134]. The seven individuals with positive or weakly positive results from Central Colombia were interpreted as false positives, because the IgA EMA test was negative. In fact, false positive results for the IgA tTG have been also reported in other ADs [6,135].

During the search, we found that some studies have reported the presence of HLA-DQ2 and HLA-DQ8 haplotype in the LA population with high titers of CD autoantibodies and diagnosis of CD (Table 4) [12,85,88,111,136–141]. HLA DQ2.5 is common in the LA population, whilst approximately 90% of studied Amerindian individuals carry the DQ8 haplotype [14,142,143].

**Table 4:** Presence of HLA in celiac disease patients.

Country	Author	Year	Comment
**Argentina**			
	Palavecino EA, *et al*. [136]	1990	95% of CD patients were positive for HLA-DQ2 versus 40% in controls
	Herrera M, *et al*. * [137]	1994	100% (n = 16) pediatric patients had DQA1*0501 and DQB1*0201
	Parada A, et al. [12]	2011	55% had HLA-DQB1*201 and 202
**Brazil**			
	Silva EM, et al. * [138]	2000	The frequency of HLA DRB1*03, HLA DRB1*07, and HLA DQB1*02 was significantly higher in 25 CD patients than in 91 healthy controls
	Martins Rde C, *et al*. [85]	2010	66.6% (n = 90) had HLA-DQ2 and, in 20%, it was detected in association with the DRB1*04 allele, and the frequency of HLA DQ2 in 14 first-degree relatives with diagnosis of CD was 78.5%.
	Castro-antunes, et al. [88]	2010	68.5% (n = 73) had HLA-DQ-2, 17.8% HLA-DQ8 and 6.8% had DQ-2 and DQ-8
	Alencar ML, *et al*.* [94]	2012	67% (n = 21) had HLA-DQ2 or -DQ8. Two of these patients were homozygous for HLA-DQB1*02 (DQ2), and one was homozygous for HLA-DQB1*0302 (DQ8).
**Chile**			
	Perez-Bravo F *et al*. [139]	1999	The proportion of DQA1*0501 in CD patients (n = 62) and in controls (n = 124) was 0.48 vs 0.169 (p<0.0005) respectively, and DQB1*0302 was 0.43 vs. 0.242 (p<0.002), and DQB1*0201 was 0.25 vs 0.125 (p<0.037)
	Parada A, *et al*. [12]	2011	55% had HLA-DQB1*201 and 202
**Cuba**			
	Cintado A, *et al*. [111]	2006	The proportion of HLA DQ2 in CD patients (n = 22) and in controls (n = 60) was 86.3% vs. 20% respectively (p<0.000004; OR: 4.32; CI: 0.09–0.5541), the proportion of DQA1*0501 in CD patients and in controls was 86.3% vs. 56.6%, and DQB1*02 was 90.2% vs. 45%. However, the proportion of HLA DQ2 in first-degree relatives (n = 54) and in controls was 70.3% vs. 20% (p<0.0009; OR: 3.52; CI: 0.1348–0.5992)
**Uruguay**			
	Poggio Favotto RC, *et al*. [140]	2001	The proportion of HLA-DQB1*0201 in CD patients (n = 17) and in controls (n = 20) was 94.1% vs 60% respectively (p<0.0172; RR: 10.7), and the proportion of HLA-DRB1*03 in CD patients and in controls was 76.5% vs 20%, respectively (p<0.0007; RR:13)
**Venezuela**			
	Landaeta *et al*. [141]	2008	100% (n = 16) exhibited HLA-DQ2 or -DQ8. Six of these patients were DQ2 (DQA1*05 DQB1*02), and six were DQ8 (DQA1*0301 DQB1*03)

* Caucasian origin.

### Study Limitations

We would like to acknowledge the limitations of our study. First, the diagnostic criteria and the screening to evaluate the presence of CD have changed over time. The autoantibodies previously evaluated are now considered obsolete. For this reason, some older studies were excluded from the statistical model. Second, in some studies, the biopsy was not performed in all antibody-positive individuals. Also, in some studies, the biopsy was not obtained from the duodenum or the interpretation was not performed with the respective histological classification. Thus, in order to obtain accurate results, data from those studies were not included in the statistical model. Third, different ELISA protocols for testing the presence of tTG were used across the studies, so the dilution of positive IgA EMA was different among our analyses. Fourth, most of the data about CD came from populations at high risk. Therefore the prevalence might be overestimated.

Data from Colombia were not considered for estimation of disease prevalence. In addition, the meta-regression has some limitations, because the country effect is a confounding factor in the analysis of populations. All the included studies did not represent the whole country’s population where they were performed.

## Conclusions

In general the prevalence of CD in LA is similar to that reported in Europeans. CD in the LA population is frequent and is primarily reported in populations and regions with Caucasian ancestry. Nevertheless, in certain countries with substantial Caucasian ancestry such as Uruguay, the prevalence is unknown.

The low prevalence reported in some regions could be explained mainly by: the lack of knowledge of the disease, low gluten consumption (which does not entail an autoimmune response), changes in quantity and quality of cereal processing, microbiota or early childhood exposure to infectious agents that could impair the natural development of the immune system (i.e., hygiene hypothesis) [144].

## Supporting Information

S1 Fig Forest plot. Prevalence of the disease for each population using the model for tTG and EMA protocol. Population: A: Healthy individuals; B: First-degree relatives of CD patients; C: T1DM patients; D: Patients with other ADs; E: Patients with other conditions.
(TIF)

S2 Fig Forest plot. Prevalence of the disease for each population using the model for autoantibodies positive and biopsy positive. Population: A: Healthy individuals; B: First-degree relatives of CD patients; C: T1DM patients; D: Patients with other ADs; E: Patients with other conditions.
(TIF)

S3 Fig Funnel plot of the model for tTG and EMA protocol. (TIF)

S4 Fig Funnel plot for autoantibodies positive and biopsy positive. (TIF)

S1 PRISMA Checklist PRISMA checklist. (DOC)

S1 Table Presence of autoantibodies positive and biopsy positive in Latin American patients. Abbreviations: AGA: Antigliadin antibodies; CD: Celiac disease; CMV: Cytomegalovirus; DGP: deamidated gliadin peptide; EBV: Epstein barr virus; EMA: anti-endomysium antibody; GFD: Gluten free diet; HCV: Hepatitis C virus; HEV: Hepatitis E virus; JRA: juvenile rheumatoid arthritis; MS: multiple sclerosis; N/A: Not available; T1DM: Type 1 Diabetes Mellitus; T2DM: Type 2 Diabetes Mellitus; tTG: anti-tissue transglutaminase antibody; RA: Rheumatoid arthritis; SLE: Systemic Lupus Erythematosus; SS: Sjögren syndrome. €: presumptive celiac disease patients.
(DOC)

S2 Table Studies with lack of fit to the final model (tTG and EMA protocol). (DOC)

S3 Table Studies with lack of fit to the final model (positive and biopsy positive). (DOC)
