# Supplementary figures and images for: Prevalence of Celiac Disease in Latin America: A Systematic Review and Meta-Regression
Source: PLoS One. 2015 May 5;10(5):e0124040. doi: 10.1371/journal.pone.0124040 (PMC4420463; doi:10.1371/journal.pone.0124040)

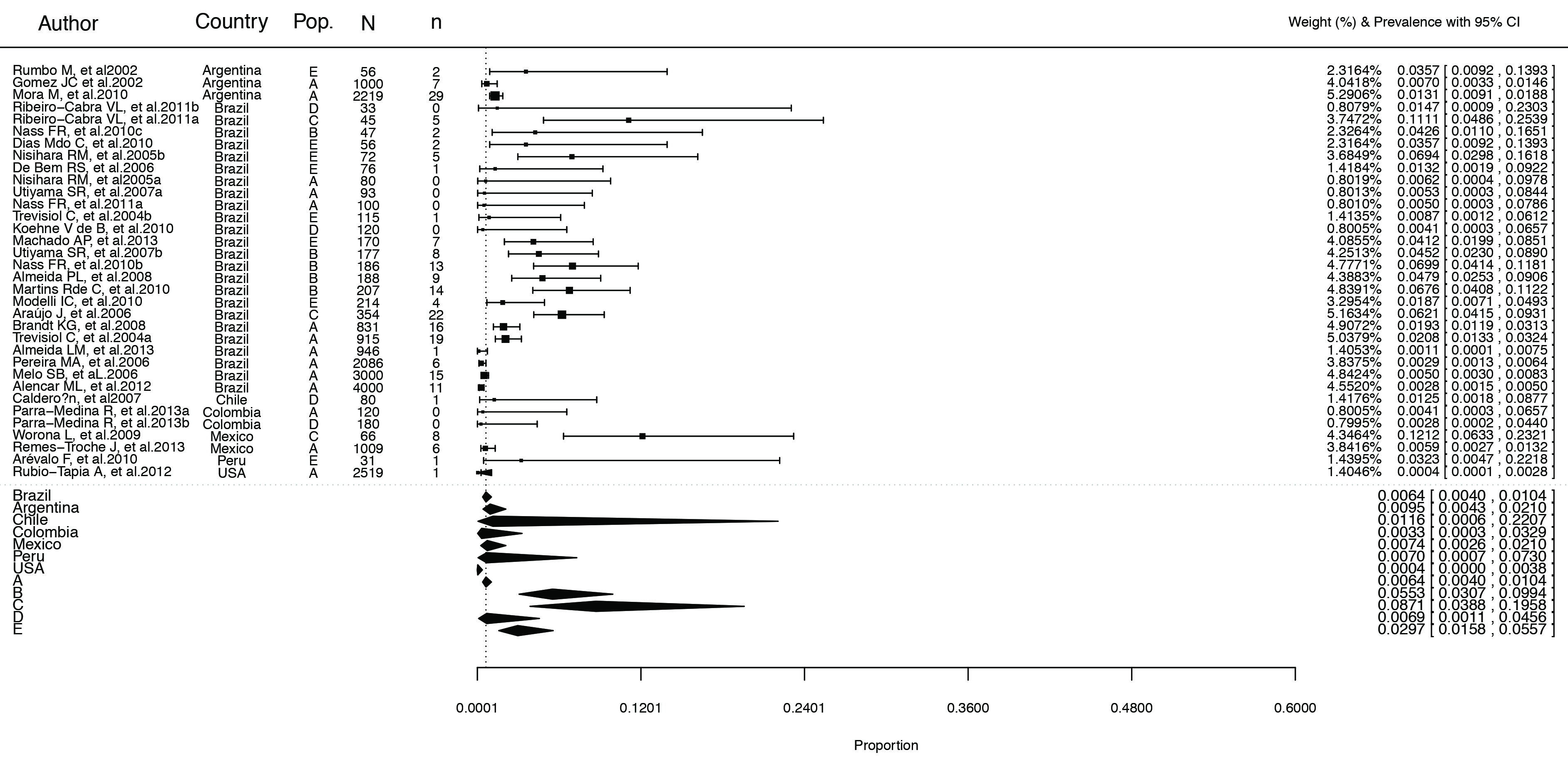

Supplement: S1 Fig — Population: A: Healthy individuals; B: First-degree relatives of CD patients; C: T1DM patients; D: Patients with other ADs; E: Patients with other conditions. (TIF) [file pone.0124040.s001.tif]

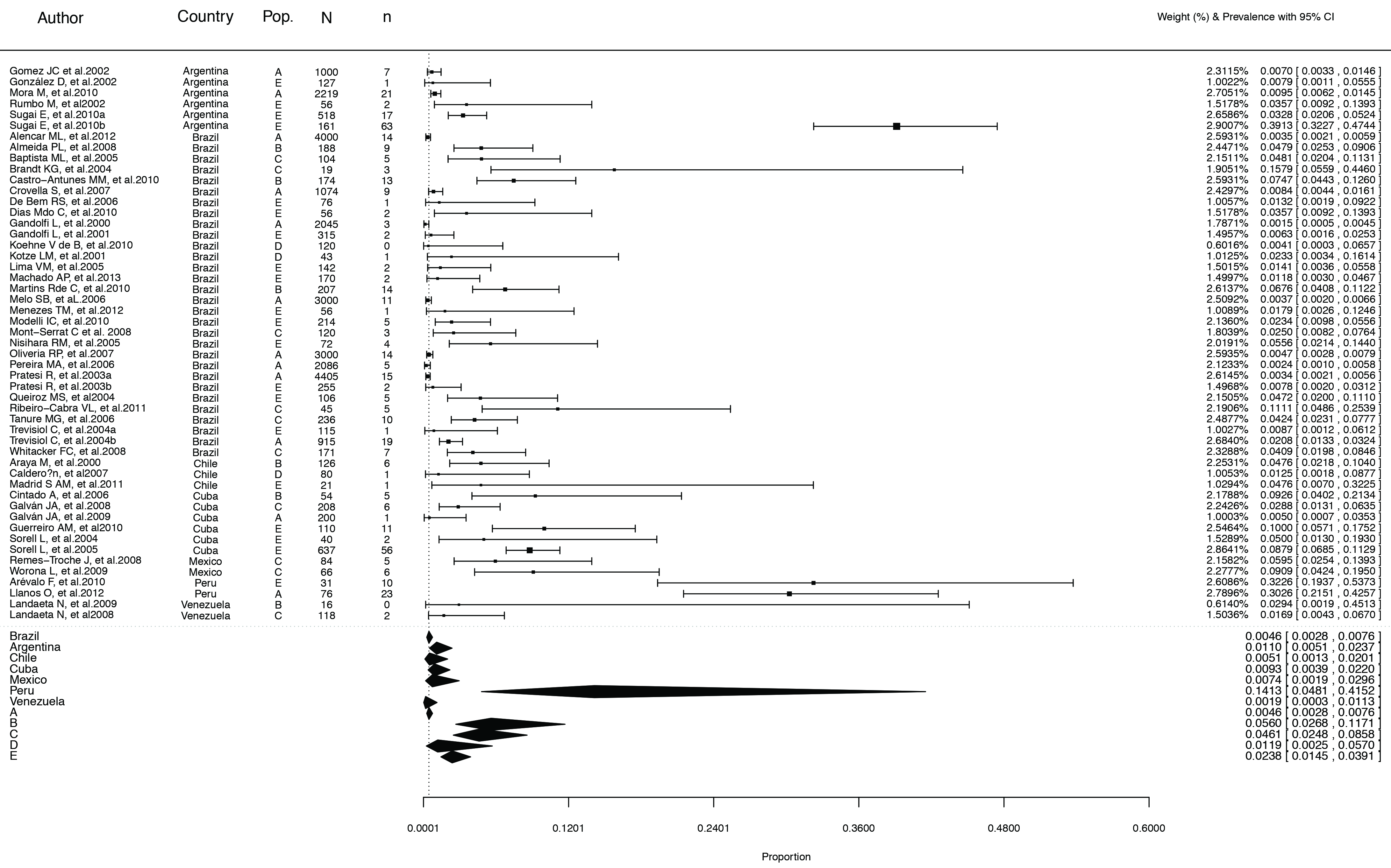

Supplement: S2 Fig — Population: A: Healthy individuals; B: First-degree relatives of CD patients; C: T1DM patients; D: Patients with other ADs; E: Patients with other conditions. (TIF) [file pone.0124040.s002.tif]

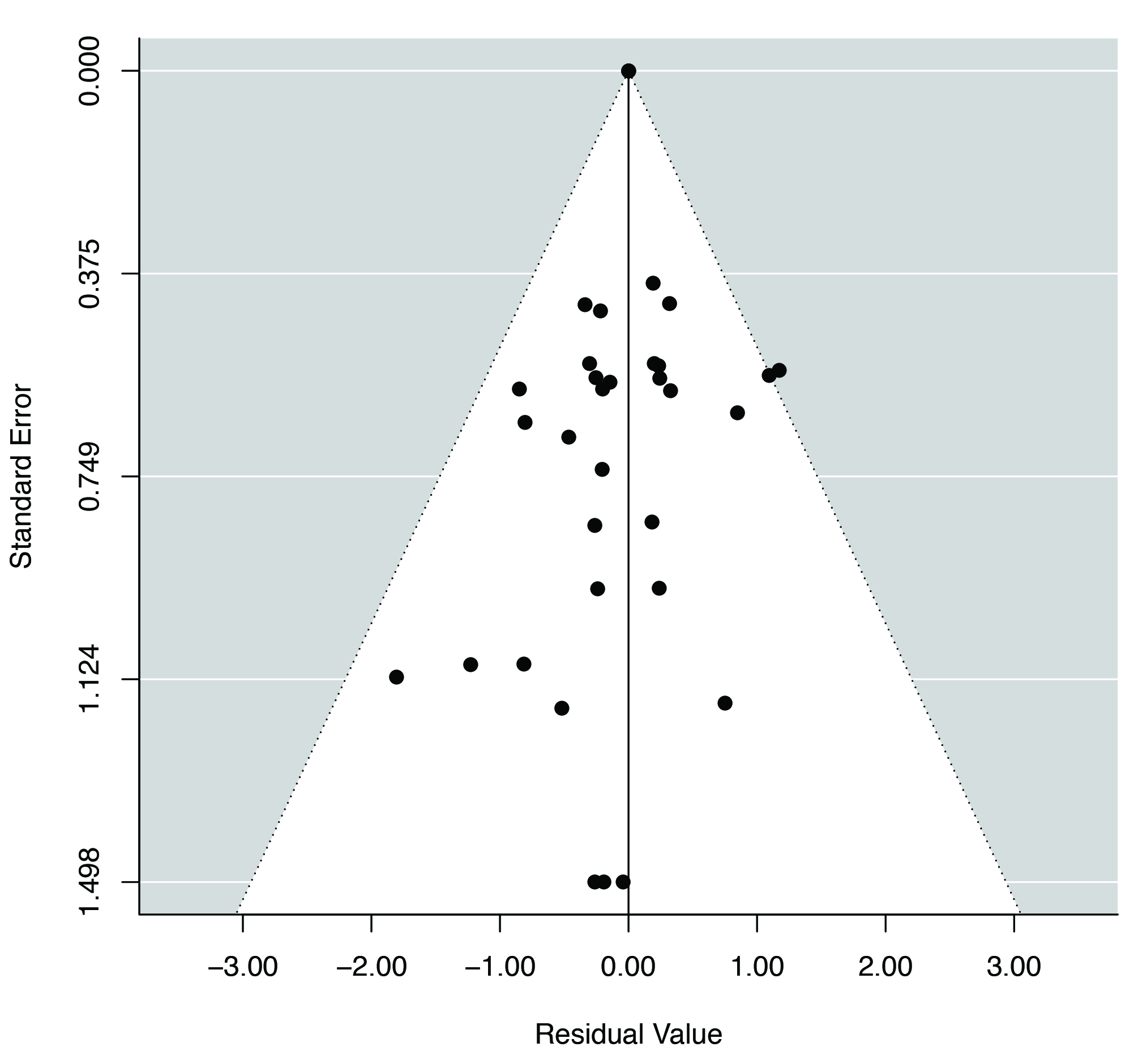

Supplement: S3 Fig — (TIF) [file pone.0124040.s003.tif]

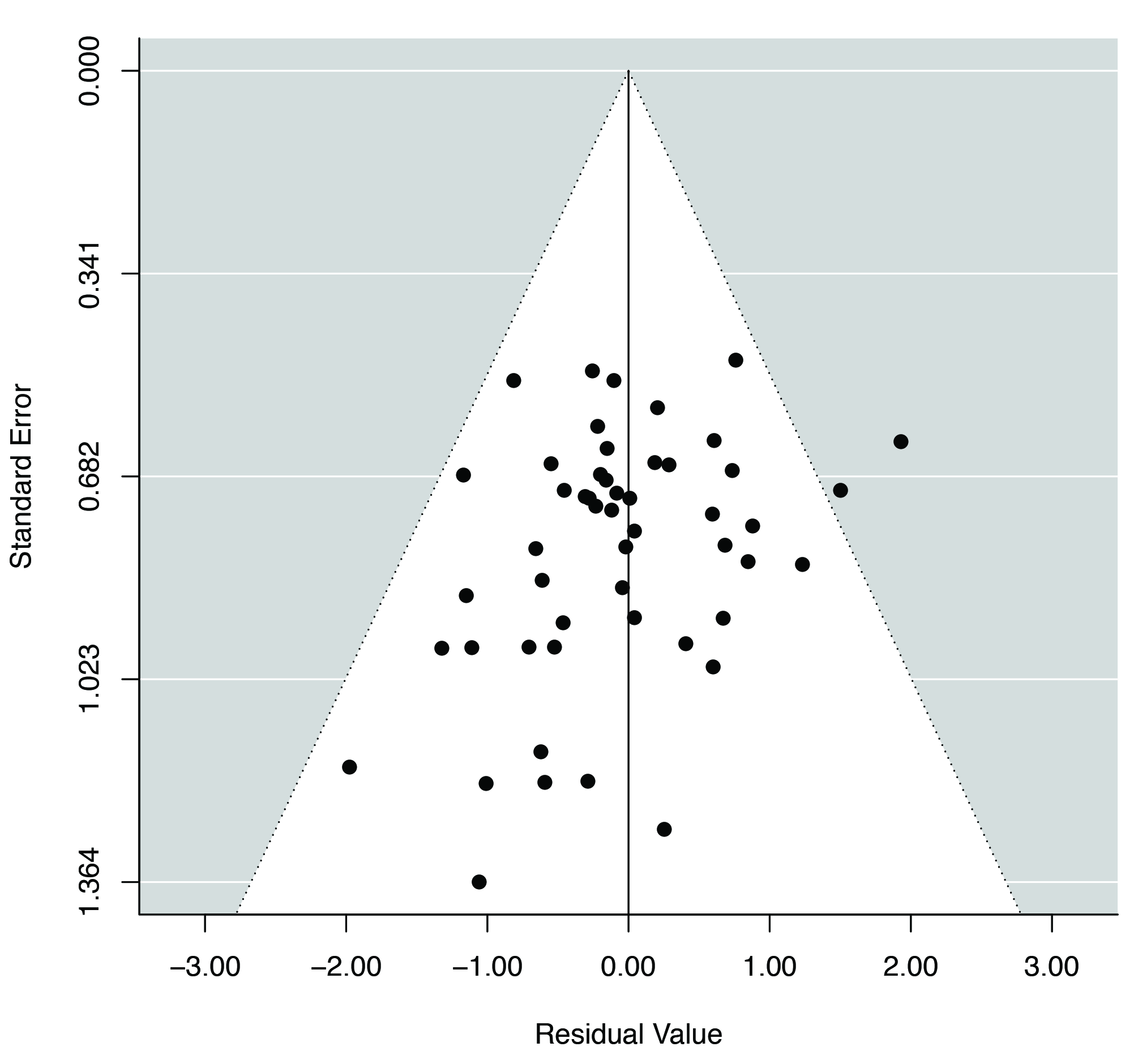

Supplement: S4 Fig — (TIF) [file pone.0124040.s004.tif]
