# Supplementary material for: Prevalence of Celiac Disease in Latin America: A Systematic Review and Meta-Regression
Source: PLoS One. 2015 May 5;10(5):e0124040. doi: 10.1371/journal.pone.0124040 (PMC4420463; doi:10.1371/journal.pone.0124040)
Supplement: S1 Table — Abbreviations: AGA: Antigliadin antibodies; CD: Celiac disease; CMV: Cytomegalovirus; DGP: deamidated gliadin peptide; EBV: Epstein barr virus; EMA: anti-endomysium antibody; GFD: Gluten free diet; HCV: Hepatitis C virus; HEV: Hepatitis E virus; JRA: juvenile rheumatoid arthritis; MS: multiple sclerosis; N/A: Not available; T1DM: Type 1 Diabetes Mellitus; T2DM: Type 2 Diabetes Mellitus; tTG: anti-tissue transglutaminase antibody; RA: Rheumatoid arthritis; SLE: Systemic Lupus Erythematosus; SS: Sjögren syndrome. €: presumptive celiac disease patients. (DOC) [file pone.0124040.s006.doc]

**S1 Table. Presence of autoantibodies positive and biopsy positive in Latin American patients**

| **Population** | **Country** | **Author** | **Year** | **Type of Study** | **Level of Evidence (Oxford)** | **Sample size** | **Age** | **Characteristic of population** | **Region** | **Screening protocol** | **Autoantibodies positives** | **Biopsy and autoantibody** |
| --- | --- | --- | --- | --- | --- | --- | --- | --- | --- | --- | --- | --- |
| **HEALTHY INDIVIDUALS** |  | | | | | | | | | | | |
|  | **Argentina** | Gomez JC *et al*. [51] | 2001 | Cross-sectional | 4 | 2000 | Adolescents and adults | Caucasian couples attending to obligatory prenuptial examination |  | AGA + EMA | 12 | 11/11 |
|  | **Argentina** | Gomez JC *et a*l. [52] | 2002 | Cross-sectional | 4 | 1000 | Adolescents and adults | Same population in the study by Gomez JC *et al.* 2001 [51] |  | AGA + EMA | 5 | 5/5 |
|  |  |  |  |  |  |  |  |  |  | tTG + EMA | 7 | 7/7 |
|  | **Argentina** | Bustos D, *et al.* [56] | 2006 | Cross-sectional | 4 | 125 | Adults | Caucasian women with least two children and without known pregnancy losses |  | tTG | 0 | NA |
|  | **Argentina** | Mora M, *et al.* [55] | 2010 | Cross-sectional | 4 | 2219 | Children and adolescents | Children and adolescents from 7 different places of residence |  | tTG + EMA | 29 | 21/22 |
|  | **Argentina** | Begué C, *et al.* [57] | 2010 | Cross-sectional | 4 | 2482 | Adults | Population of affiliates to the “Hospital Italiano de Buenos Aires" |  | tTG | 102 | NA |
|  | **Brazil** | Gandolfi L, *et al.* [59] | 2000 | Cross-sectional | 4 | 2045 | Adults | Blood donors | Central-West | AGA + EMA | 3 | 3/3 |
|  | **Brazil** | Kotze LM, *et al.* [61] | 2001 | Cross-sectional | 4 | 126 | All ages | Healthy individuals | Southern | EMA | 1 | NA |
|  | **Brazil** | Pratesi R, *et al.* [62] | 2003 | Cross-sectional | 4 | 4405 | All ages | Blood donors | Central-West | EMA | 16 | 15/16 |
|  | **Brazil** | Trevisiol C, *et al.* [66] | 2004 | Cross-sectional | 4 | 915 | Children and adolescents | Healthy individuals | North-eastern | tTG + EMA | 19 | 19/19 |
|  | **Brazil** | Baptista ML, *et al.* [67] | 2004 | Cross-sectional | 4 | 105 | Children and adolescents | Healthy individuals | Southern | EMA | 0 | NA |
|  | **Brazil** | Nisihara RM, et al [68] | 2005 | Cross-sectional | 4 | 80 | All ages | Healthy individuals | Southern | tTG + EMA | 0 | NA |
|  | **Brazil** | Pereira MA, *et al.* [71] | 2006 | Cross-sectional | 4 | 2086 | Adults | Blood donors with normal serological tests for HIV, HBV, HCV and liver enzymes | Southern | tTG + EMA | 6 | 5/6 |
|  | **Brazil** | Melo SB, *et al.* [72] | 2006 | Cross-sectional | 4 | 3000 | Adults | Blood donors | Southeastern | tTG + EMA | 15 | 11/13 |
|  | **Brazil** | Crovella S, *et al.* [75] | 2007 | Cross-sectional | 4 | 1074 | Adolescents and adults | University students and subjects were coming from sub-urban impoverished areas | North-eastern | tTG | 9 | 9/9 |
|  | **Brazil** | Nisihara RM, *et al* [76] | 2007 | Cross-sectional | 4 | 97 | Adults | Healthy individuals | Southern | EMA | 0 | NA |
|  | **Brazil** | Oliveria RP, *et al.* [77] | 2007 | Cross-sectional | 4 | 3000 | Adults | Blood donors of healthy volunteers | Southeastern | tTG | 45 | 14/21 |
|  | **Brazil** | Utiyama SR, *et al.* [78] | 2007 | Cross-sectional | 4 | 93 | All ages | Healthy individuals | Southern | tTG + EMA | 0 | NA |
|  | **Brazil** | Brandt KG, *et al.* [82] | 2008 | Cross-sectional | 4 | 831 | Children and adolescents | Children and adolescents apparently healthy | North-eastern | tTG + EMA | 16 | NA |
|  | **Brazil** | Utiyama SR, *et al.* [84] | 2010 | Cross-sectional | 4 | 501 | All ages | Indigenous (320) and non-Indigenous healthy individuals (180) | Southern | EMA | 0 | NA |
|  | **Brazil** | Nass FR, *et al.* [89] | 2011 | Cross-sectional | 4 | 100 | All ages | Healthy volunteers not related to CD patients | Southern | tTG + EMA | 0 | NA |
|  | **Brazil** | Goeldner I, et al. [91] | 2011 | Cross-sectional | 4 | 100 | Adults | Healthy individuals | Southern | EMA | 0 | NA |
|  | **Brazil** | Nisihara R, *et al.* [92] | 2011 | Cross-sectional | 4 | 97 | Adults | Healthy individuals | Southern | EMA | 0 | NA |
|  | **Brazil** | Andretta MA, *et al.* [93] | 2012 | Cross-sectional | 4 | 57 | Adults | Healthy individuals | Southern | EMA | 0 | NA |
|  | **Brazil** | Alencar ML, *et al.* [94] | 2012 | Cross-sectional | 4 | 4000 | Adults | Donors had to have a fixed residence for at least two years in the city of Sao Paulo. Individuals with known CD were excluded from participation. | Southeastern | tTG + EMA | 11 | 14/21 |
|  | **Brazil** | Almeida RC, *et al.* [95] | 2012 | Cross-sectional | 4 | 860 | All ages | Sub-Saharan African-derived Brazilian communities | Northeastern | EMA | 0 | NA |
|  | **Brazil** | Almeida LM, *et al.* [97] | 2013 | Cross-sectional | 4 | 946 | Adults | Individuals over 60 years | Central-West | tTG + EMA | 1 | NA |
|  | **Brazil** | Skare T, *et al.* [98] | 2013 | Cross-sectional | 4 | 100 | Adults | Healthy individuals | Southern | EMA | 0 | NA |
|  | **Colombia** | Parra-Medina R, *et al. (Present study)* | 2013 | Cross-sectional | 4 | 120 | Adults | Healthy individuals | Central | tTG + EMA | 0 | NA |
|  |  | Parra-Medina R, *et al. (Present study)* | 2013 | Cross-sectional | 4 | 140 | Adults | Healthy individuals | Northwest | tTG | 0 | NA |
|  | **Cuba** | Galván JA, *et al.* [105] | 2005 | Cross-sectional | 4 | 595 | Children | Apparently healthy |  | tTG | 7 | NA |
|  | **Cuba** | Cintado A, *et al.* [111] | 2006 | Cross-sectional | 4 | 60 | All ages | Healthy individuals |  | tTG | 0 | NA |
|  | **Cuba** | Galván JA, *et al.* [109] | 2009 | Cross-sectional | 4 | 200 | Adolescents and adults | Apparently healthy |  | tTG | 1 | 1/1 |
|  | **Cuba** | Sarmiento L, *et al.* [112] | 2012 | Cross-sectional | 4 | 164 | Children and adolescents | Samples without viral infections (HEV, EBV, CMV, HCV) |  | tTG | 0 | NA |
|  | **Mexico** | Madrazo de la Garza JA, *et al.* [113] | 2006 | Cross-sectional | 4 | 1000 | Adolescents and adults | College students |  | tTG | 16 | NA |
|  | **Mexico** | Remes-Troche J, *et al.* [114,117] | 2013 | Cross-sectional | 4 | 1009 | Adolescents and adults | Serum positive for IgA tTG in previous study of Remes-Troche *et al.* 2006 (97) |  | tTG + EMA | 6 | NA |
|  | **Peru** | Llanos O, *et al.* [119] | 2012 | Retrospective, observational | 4 | 76 | Adults | Patients over 18 years |  | tTG | 39 | 23/39 |
|  | **USA** | Rubio-Tapia A, *et al.* [122] | 2012 | Cross-sectional | 4 | 2519 | All ages | Hispanics residents in USA |  | tTG + EMA | 1 | NA |
| **FIRST DEGREE RELATIVES OF CELIAC DISEASE PATIENTS** |  | | | | | | | | | | | |
|  | **Brazil** | Utiyama SR, *et al.* [78] | 2007 | Cross-sectional | 4 | 177 | All ages |  | Southern | tTG + EMA | 8 | NA |
|  | **Brazil** | Almeida PL, *et al* [80] | 2008 | Cross-sectional | 4 | 188 | All ages |  | Central-West | tTG + EMA | 9 | 9/9 |
|  | **Brazil** | Martins Rde C, *et al.* [85] | 2010 | Cross-sectional | 4 | 207 | All ages |  | Central-West | tTG + EMA | 14 | 14/14 |
|  | **Brazil** | Castro-Antunes MM, *et al.*  [88] | 2010 | Cross-sectional | 4 | 174 | Adults |  | North-eastern | tTG | 34 | 13/22 |
|  | **Brazil** | Nass FR, *et al.* [89] | 2010 | Cross-sectional | 4 | 186 | All ages | Serum samples from relatives of CD patients were collected between 1997 and 2000 | Southern | tTG + EMA | 13 | NA |
|  | **Brazil** | Nass FR, *et al.* [89] | 2010 | Cross-sectional | 4 | 47 | All ages | Serum samples from relatives of CD patients were collected between 2006 and 2007 | Southern | tTG + EMA | 2 | NA |
|  | **Chile** | Araya M, *et al.* [99] | 2000 | Cross-sectional | 4 | 126 | All ages |  |  | EMA | 6 | 6/6 |
|  | **Cuba** | Cintado A, *et al.* [111] | 2006 | Cross-sectional | 4 | 54 | All ages |  |  | tTG | 10 | 5/7 |
|  | **Venezuela** | Landaeta N, *et al.* [121] | 2009 | Cross-sectional | 4 | 16 | Adolescents and adults |  |  | tTG + EMA | 5 | 0/2 |
|
| **TYPE 1 DIABETES MELLITUS PATIENTS** |  | | | | | | | | | | | |
|  | **Brazil** | Brandt KG, *et al.* [64] | 2004 | Cross-sectional | 4 | 19 | Children and adolescents |  | Southern | tTG | 4 | 3/4 |
|
|
|
|  | **Brazil** | Baptista ML, *et al.* [67] | 2005 | Cross-sectional | 4 | 104 | Children and adolescents |  | Southern | EMA | 9 | 5/9 |
|  | **Brazil** | Tanure MG, *et al* [70] | 2006 | Cross-sectional | 4 | 236 | Children and adolescents |  | Southeastern | AGA + EMA | 5 | 10/19 |
|  | **Brazil** | Araújo J, et al. [74] | 2006 | Cross-sectional | 4 | 354 | Children and adolescents |  | Northeastern | tTG + EMA | 22 | NA |
|  | **Brazil** | Whitacker FC, *et al.* [79] | 2008 | Cross-sectional | 4 | 171 | Children and adolescents |  | Southeastern | EMA | 9 | 7/9 |
|  | **Brazil** | Mont-Serrat C *et al.* [81] | 2008 | Cross-sectional | 4 | 120 | Children and adolescents |  | Southeastern | tTG | 3 | 3/3 |
|  | **Brazil** | Ribeiro-Cabra VL, *et al.* [90] | 2011 | Cross-sectional | 4 | 45 | Adolescents and adults |  | Southeastern | tTG + EMA | 5 | 5/5 |
|  | **Colombia** | Parra-Medina R, *et al. (Present study)* | 2013 | Cross-sectional | 4 | 67 | Children and adolescents |  | Northwest | tTG | 0 | NA |
|  | **Cuba** | Castañeda C, *et al* [103] | 2004 | Cross-sectional | 4 | 247 | Children and adolescents |  |  | tTG | 6 | NA |
|  | **Cuba** | Galván JA, *et al* [107] | 2008 | Cross-sectional | 4 | 208 | All ages |  |  | tTG | 14 | 6/208 |
|  | **Mexico** | Remes-Troche J, *et al.* [115] | 2008 | Cross-sectional | 4 | 84 | Adults | Mexican Mestizo patients |  | tTG | 9 | 5/7 |
|  | **Mexico** | Worona L, *et al* [116]*.* | 2009 | Cross-sectional | 4 | 66 | Children and adolescents |  |  | tTG + EMA | 8 | 6/8 |
|  | **Venezuela** | Landaeta N, *et al* [120] | 2008 | Cross-sectional | 4 | 118 | Children and adolescents |  |  | tTG | 4 | 2/3 |
| **PATIENTS WITH CLINICAL SUSPICION OF CELIAC DISEASE** |  | | | | | | | | | | | |
|  | **Brazil** | Gandolfi L, *et al.* [60] | 2001 | Cross-sectional | 4 | 315 | Children | Patients with different types of malnutrition | Central-West | EMA | 2 | 2/2 |
|  | **Brazil** | Queiroz MS, *et al* [65] | 2004 | Cross-sectional | 4 | 106 | Children | Patients with short stature who have no gastrointestinal symptoms | Southeastern | EMA | 6 | 5/6 |
|  | **Brazil** | Lima VM, *et al.* [68] | 2005 | Cross-sectional | 4 | 142 | Adolescents and adults | Dyspeptic patients | Central-West | AGA + EMA | 2 | 2 |
|  | **Brazil** | Modelli IC, *et al* [87] | 2010 | Cross-sectional | 4 | 214 | Adolescents and adults | Symptomatic children | Central-West | AGA + EMA | 4 | 5/5 |
|  |  |  |  |  | 4 |  |  |  |  | tTG + EMA | 4 | 5/5 |
|  | **Cuba** | Sorell L, *et al.* [104] | 2005 | Cross-sectional | 4 | 637 | All ages | Patients with AGA positive and suggestive biopsy |  | tTG | 88 | 56/88 |
|  | **Cuba** | Santana-Porbén S, *et al.* [108] | 2009 | Cross-sectional | 4 | 728 | NA | Patients with malnutrition |  | Scoring system composed of an interrogation, antibody assays (AGA and tTG), biopsy, nutritional status and response to GFD. | 28 € | NA |
|  | **Cuba** | Guerreiro AM, *et al.* [110] | 2010 | Cross-sectional | 4 | 110 | Children | Patients with symptoms suggestive of CD |  | AGA or tTG | 23 | 11/23 |
|  | **Peru** | Arévalo F, *et al.* [118] | 2010 | Cross-sectional | 4 | 31 | Adults | Patients with suggestive biopsy |  | AGA + EMA | 6 | 10/31 |
|  |  |  |  |  |  |  |  |  |  | tTG + EMA | 1 | NA |
| **PATIENTS WITH OTHER AUTOIMMUNE DISEASE** |  | | | | | | | | | | | |
|  | **Brazil** | Kotze LM, *et al.* [61] | 2001 | Cross-sectional | 4 | 43 | All ages | Crohn’s disease and colitis ulcerative patients | Southern | EMA | 1 | 1/1 |
|  | **Brazil** | Nisihara RM, *et al.* [76] | 2007 | Cross-sectional | 4 | 85 | Adults | RA patients | Southern | EMA | 0 | NA |
|  | **Brazil** | Koehne V de B, *et al.* [83] | 2010 | Cross-sectional | 4 | 190 | All ages | SLE (69), RA (48), JRA (32) and spondyloarthropathy (41) patients | Southeastern | tTG + EMA | 0 | 0/18 |
|  | **Brazil** | Ribeiro-Cabra VL, *et al.* [90] | 2011 | Cross-sectional | 4 | 33 | Adults | Crohn’s disease patients | Southeastern | tTG + EMA | 0 | NA |
|  | **Brazil** | Goeldner I, *et al.* [91] | 2011 | Cross-sectional | 4 | 156 | Adults | RA patients | Southeastern | EMA | 0 | NA |
|  | **Brazil** | Nisihara R, *et al* [92] | 2011 | Cross-sectional | 4 | 105 | Adults | Scleroderma patients | Southern | EMA | 0 | NA |
|  | **Brazil** | Andretta MA, *et al.* [93] | 2012 | Cross-sectional | 4 | 70 | Adults | Spondyloarthritis patients | Southern | EMA | 0 | NA |
|  | **Brazil** | Skare T, *et al.* [98] | 2013 | Cross-sectional | 4 | 380 | Adults | RA (156), scleroderma (105), spondyloarthropathy (70), and JRA (49) patients | Southern | EMA | 0 | NA |
|  | **Chile** | Calderón HP, *et al* [100] | 2007 | Cross-sectional | 4 | 80 | Adolescents and adults | Psoriasis patients |  | tTG + EMA | 1 | 1/1 |
|  | **Colombia** | Parra-Medina R, *et al. (Present study)* | 2013 | Cross-sectional | 4 | 180 | Adults | SLE (60), RA (60) and SS (60) patients | Central | tTG + EMA | 0 | NA |
|  | **Colombia** | Parra-Medina R, *et al. (Present study)* | 2013 | Cross-sectional | 4 | 459 | Adults | SLE (119), RA (151), SS (82) and MS (98) | Northwest | tTG | 0 | NA |
|  | **Cuba** | Sorell L, *et al.* [104] | 2005 | Cross-sectional | 4 | 100 | All ages | Autoimmune thyroid disease patients |  | tTG | 3 | NA |
|  | **Cuba** | Sánchez JC, *et al.* [106] | 2007 | Cross-sectional | 4 | 142 | Adults | Latent autoimmune diabetes of adult patients |  | tTG | 5 | NA |
| **OTHERS CONDITIONS** |  | | | | | | | | | | | |
|  | **Argentina** | González D, *et al*. [53] | 2002 | Cross-sectional | 4 | 127 | Adults | Osteoporotic patients |  | AGA + EMA | 1 | 1/1 |
|  | **Argentina** | Rumbo M, *et al* [54] | 2002 | Cross-sectional | 4 | 56 | Children and adolescents | Down´s syndrome patients |  | tTG + EMA | 2 | 2/2 |
|  | **Argentina** | Bustos D, *et al.* [56] | 2006 | Cross-sectional | 4 | 118 | Adults | Caucasian women with recurrent pregnancy loss in the first trimester |  | tTG | 3 | NA |
|  | **Argentina** | Sugai E, *et al.* [58] | 2010 | Cross-sectional | 4 | 161 | Adults | High risk population |  | NA | NA | 63 |
|  | **Argentina** | Sugai E, *et al.* [58] | 2010 | Cross-sectional | 4 | 518 | Adults | Low risk population |  | NA | NA | 17 |
|  | **Brazil** | Kotze LM, *et al.* [61] | 2001 | Cross-sectional | 4 | 51 | All ages | Other gastrointestinal diseases | Southern | EMA | 0 | NA |
|  | **Brazil** | Pratesi R, *et al.* [63] | 2003 | Cross-sectional | 4 | 255 | All ages | Epileptic patients | Central-West | EMA | 2 | 2/2 |
|  | **Brazil** | Trevisiol C, *et al.* [66] | 2004 | Cross-sectional | 4 | 115 | Children and adolescents | Patients with gynecological illness | North-eastern | tTG + EMA | 1 | 1/1 |
|  | **Brazil** | Nisihara RM, *et al.* [69] | 2005 | Cross-sectional | 4 | 72 | Children and adolescents | Down´s syndrome patients | Southern | tTG + EMA | 5 | 4/5 |
|  | **Brazil** | De Bem RS, *et al.* [73] | 2006 | Cross-sectional | 4 | 76 | Adults | Precardiac transplant patients with advanced cardiomyopathy | Southern | tTG + EMA | 1 | 1/1 |
|  | **Brazil** | Dias Mdo C, *et al.* [86] | 2010 | Cross-sectional | 4 | 56 | All ages | Turner syndrome patients | Central-West | tTG + EMA | 2 | 2/2 |
|  | **Brazil** | Goeldner I, et al. [91] | 2011 | Cross-sectional | 4 | 200 | All ages | Relatives of RA patients | Southern | EMA | 2 | NA |
|
|  | **Brazil** | Menezes TM, *et al.* [96] | 2012 | Cross-sectional | 4 | 56 | Children and adolescents | Myocarditis and dilated cardiomyopathy patients | North-eastern | tTG | 1 | 1/1 |
|  | **Brazil** | Machado AP, *et al.* [50] | 2013 | Cross-sectional | 4 | 170 | NA | Infertility woman | Northeastern | tTG + EMA | 7 | 2/7 |
|  | **Chile** | Madrid SA, *et al.* [101] | 2011 | Cross-sectional | 4 | 21 | Adults | Cryptogenic epilepsy patients |  | tTG + DGP | 1 | 1/1 |
|  | **Colombia** | Parra-Medina R, et al. (Present study) | 2013 | Cross-sectional | 4 | 24 |  | T1DM Relatives | Northwest | tTG | 1 | NA |
|  | **Cuba** | Sorell L, *et al.* [102] | 2004 | Cross-sectional | 4 | 40 | All ages | Giardiasis patients |  | AGA + EMA | 2 | 2/2 |
|  | **Cuba** | Castañeda C, *et al.* [103] | 2004 | Cross-sectional | 4 | 263 | NA | Down´s syndrome |  | tTG | 6 | NA |
|
|  | **Cuba** | Sorell L, *et al.* [104] | 2005 | Cross-sectional | 4 | 115 | All ages | Hypertransaminasemia |  | tTG | 1 | NA |
|  |  |  |  |  |  |  |  |  |  |  |  |  |
|  | **Cuba** | Sánchez JC, *et al.* [106] | 2007 | Cross-sectional | 4 | 142 | Adults | Patients with T2DM |  | tTG | 4 | NA |
|  | **Cuba** | Sarmiento L, *et al.* [112] | 2012 | Cross-sectional | 4 | 82 | All ages | Samples with viral infections (HEV, EBV, CMV, HCV) |  | tTG | 20 | NA |

Abbreviations: AGA: Antigliadin antibodies; CD: Celiac disease; CMV: Cytomegalovirus; DGP: deamidated gliadin peptide; EBV: Epstein barr virus; EMA: anti-endomysium antibody; GFD: Gluten free diet; HCV: Hepatitis C virus; HEV: Hepatitis E virus; JRA: juvenile rheumatoid arthritis; MS: multiple sclerosis; N/A: Not available; T1DM: Type 1 Diabetes Mellitus; T2DM: Type 2 Diabetes Mellitus; tTG: anti-tissue transglutaminase antibody; RA: Rheumatoid arthritis; SLE: Systemic Lupus Erythematosus; SS: Sjögren syndrome.

€: presumptive celiac disease patients
