# Supplementary material for: Prevalence of Celiac Disease in Latin America: A Systematic Review and Meta-Regression
Source: PLoS One. 2015 May 5;10(5):e0124040. doi: 10.1371/journal.pone.0124040 (PMC4420463; doi:10.1371/journal.pone.0124040)
Supplement: S2 Table — (DOC) [file pone.0124040.s007.doc]

**S2 Table: Studies with lack of fit to the final model (tTG and EMA protocol)**

| Author | Country | Population | Sample size | Autoantibody | prop |
| --- | --- | --- | --- | --- | --- |
| Brandt KG, *et al.* 2008 [64] | Brazil | A | 831 | 16 | 1.925391 |
| Trevisiol C, *et al.* 2004a [66] | Brazil | A | 915 | 19 | 2.076503 |
