# Supplementary material for: Prevalence of Celiac Disease in Latin America: A Systematic Review and Meta-Regression
Source: PLoS One. 2015 May 5;10(5):e0124040. doi: 10.1371/journal.pone.0124040 (PMC4420463; doi:10.1371/journal.pone.0124040)
Supplement: S3 Table — (DOC) [file pone.0124040.s008.doc]

**S3 Table: Studies with lack of fit to the final model (positive and biopsy positive)**

| Author | Country | Population | Sample size | Biopsy and autoantibody | prop |
| --- | --- | --- | --- | --- | --- |
| Sugai E, *et al.* 2010b [58] | Argentina | E | 161 | 63 | 39.130435 |
| Trevisiol C, *et al.* 2004b [56] | Brazil | A | 915 | 19 | 2.076503 |
